# Clinical features of acquired erythrocytosis: Low levels of serum erythropoietin in a subset of non‐neoplastic erythrocytosis patients

**DOI:** 10.1002/cam4.4958

**Published:** 2022-07-01

**Authors:** Yosuke Mori, Marito Araki, Soji Morishita, Misa Imai, Yoko Edahiro, Masafumi Ito, Tomonori Ochiai, Shuichi Shirane, Yoshinori Hashimoto, Hajime Yasuda, Jun Ando, Miki Ando, Norio Komatsu

**Affiliations:** ^1^ Department of Hematology Juntendo University Graduate School of Medicine Tokyo Japan; ^2^ Laboratory for the Development of Therapies Against MPN Juntendo University Graduate School of Medicine Tokyo Japan; ^3^ Department of Advanced Hematology Juntendo University Graduate School of Medicine Tokyo Japan; ^4^ Department of Pathology Japanese Red Cross Aichi Medical Center Nagoya Daiichi Hospital Nagoya Japan; ^5^ Department of Cell Therapy and Transfusion Medicine Juntendo University Graduate School of Medicine Tokyo Japan; ^6^ PharmaEssentia Japan KK Tokyo Japan

**Keywords:** erythrocytosis, myeloproliferative neoplasms, erythropoietin, drinking habit, JAK2, lifestyle‐related disease

## Abstract

**Background:**

Acquired erythrocytosis can be classified into polycythemia vera (PV) and non‐neoplastic erythrocytosis (NNE). The vast majority of PV patients harbor *JAK2* mutations, but differentiating *JAK2* mutation‐negative PV from NNE is challenging due to a lack of definitive molecular markers.

**Methods:**

We studied the clinical features of 121 patients with erythrocytosis of which 47 (38.8%) were JAK2 mutation‐positive and also fulfilled the diagnostic criteria for PV, and 67 (55.4%) JAK2 mutation‐negative erythrocytosis patients who were diagnosed as NNE. Diagnosis was strictly based on driver mutation analysis and central pathology review.

**Results:**

No *JAK2* mutation‐negative PV patients were found in our cohort. The NNE group showed significantly younger (*p* < 0.01) age with higher frequency of smoking (*p* < 0.001), alcohol consumption (*p* < 0.001), and diabetes mellitus (*p* < 0.05), whereas the PV group (*n* = 47) showed significantly higher white blood cell count, platelet count, and lactate dehydrogenase (*p* < 0.001). Although serum erythropoietin (EPO) levels were significantly higher in NNE compared to PV (*p* < 0.001), approximately 40% of the NNE patients had EPO levels below the lower range of normal, fulfilling a minor diagnostic criterion of PV and raising the possibility of PV misdiagnosis.

**Conclusion:**

Low EPO levels in *JAK2* mutation‐negative erythrocytosis may not be a reliable diagnostic criterion for distinguishing PV from NNE.

## Graphical abstract

Heat maps illustrating lifestyle habits and pre‐existing clinical conditions at the time of initial diagnosis in non‐neoplastic erythrocytosis (NNE) and polycythemia vera patients. Black, white, and gray indicate presence, absence, and unknown status, respectively. The NNE group showed a higher frequency of smoking, alcohol consumption, and type 2 diabetes mellitus.

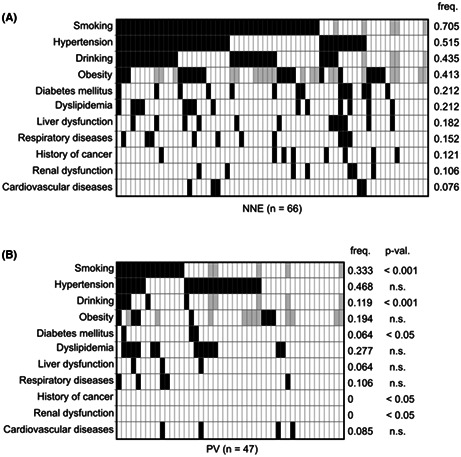

## 1 INTRODUCTION

Erythrocytosis can be classified into three disease entities: (1) Congenital/hereditary erythrocytosis caused by an inheritance of genetic traits promoting erythropoietin (EPO) signaling or production; (2) polycythemia vera (PV), a hematological malignancy caused by the acquisition of somatic mutations in hematopoietic stem/progenitor cells promoting erythropoiesis; and (3) non‐neoplastic erythrocytosis (NNE), which includes reactive erythrocytosis, relative erythrocytosis, and idiopathic erythrocytosis.^1^ Accurate diagnosis and discrimination of these entities, especially concerning PV, is critical for providing appropriate treatment.

More than 95% of patients with PV present with disease‐defining mutations such as *JAK2* V617F and *JAK2* exon 12,^2^ and the presence of these mutations has been adopted as a major criterion in the diagnostic criteria for PV by the World Health Organization.^3^ However, a subgroup of PV patients with low EPO levels, whose bone marrow specimen exhibit PV features, test negative for *JAK2* mutations.^3^, ^4^ Of these, non‐canonical *JAK2* mutations and infrequent mutations in genes that may promote erythrocytosis have been reported.^4^, ^5^, ^6^ However, because the pathogenesis is unknown in most cases, the clinical implications of *JAK2*‐negative PV and whether this entity really exists remain ambiguous.^4^, ^5^, ^6^, ^7^ Furthermore, the clinical features differentiating NNE from PV have not been extensively studied.

Reactive erythrocytosis is generally defined by ruling out PV and confirming elevated serum EPO levels. Defining relative erythrocytosis is challenging in the real‐world setting because laboratory tests for measuring red cell mass are not routinely available. In addition, discrimination between relative erythrocytosis and idiopathic erythrocytosis is also difficult because of the unclear pathogenesis and lack of definitive markers for idiopathic erythrocytosis. The fact that diagnosis of *JAK2*‐negative PV essentially depends on bone marrow pathology makes differentiating between *JAK2*‐negative PV, relative erythrocytosis, and idiopathic erythrocytosis difficult. Thus, this study aimed to evaluate patients presenting with acquired erythrocytosis and to identify distinct clinical characteristics of NNE that distinguish it from PV.

## 2 METHODS

### 2.1 Patients

We included patients who visited Juntendo University Hospital and its affiliated institutions between January 2010 and December 2019 and were suspected of having myeloproliferative neoplasms (MPNs). Screening for chronic myeloid leukemia (CML) was performed by peripheral blood fluorescence in situ hybridization, bone marrow G‐band analysis, or bone marrow real‐time polymerase chain reaction (PCR). Congenital erythrocytosis was screened based on the presence of a family history. This study was approved by the ethics committee of the School of Medicine, Juntendo University (IRB#M12‐0895) and was conducted in accordance with the 1975 Helsinki Declaration. Written informed consent was obtained prior to the use of the samples and the collection of clinical records.

### 2.2 Driver mutation and *CREB3L1* expression analysis

*JAK2* V617F, *MPL* exon 10, and *CALR* exon 9 mutations were analyzed as previously described.^8^, ^9^, ^10^ Briefly, detection of *JAK2* V617F was first performed by ABC‐PCR, and in negative patients, allele‐specific quantitative PCR was further conducted to confirm negativity. For detection of *MPL* exon 9 and *CALR* exon 9 mutations, two sets of primers (common forward: AGT AGG GGC TGG CTG GAT, W515L‐specific forward: GGC CTG CTG CTG CTG AAG TT, W515K‐specific reverse: (FAM)‐ACC TGT AGT GTG CAG GAA ACT TCT T, and common reverse: (FAM)‐GGT CAC AGA GCG AAC CAA GA) and a set of primers (forward: (FAM)‐TGG TCC TGG TCC TGA TGT C and reverse: GGA ACA AAA CCA AAA TCC AC) were used to amplify the DNA fragment containing *MPL* exon 10 and *CALR* exon 9, respectively, and the amplicons were subsequently applied to capillary electrophoresis. For patients who were negative for these mutations, *JAK2* exon 12 mutations were further screened using Sanger sequencing.^11^ *CREB3L1* mRNA levels in peripheral blood platelets (PLT) were measured by SYBR green‐based reverse transcription‐quantitative PCR with a set of primers (forward: GGA GAA TGC CAA CAG GAC, and reverse: ACC AGA ACA AAG CAC AAG G) as previously described.^12^

### 2.3 Data collection

Medical history, smoking habits, and weight at the time of first visit were collected from medical records. Heavy drinking was defined as daily consumption of ≥40 g of ethanol. Obesity was defined as body mass index (BMI) of ≥25 kg/m^2^. Splenic enlargement was evaluated by palpation, abdominal ultrasonography, or abdominal computed tomography, and splenomegaly was diagnosed by the attending physician. End‐stage renal disease patients receiving regular EPO injections were included in the study but were excluded from the EPO analysis.

### 2.4 Statistical analysis

Comparisons between the NNE and PV groups were performed using Fisher's exact test for categorical variables and the Mann–Whitney *U* test for continuous variables. All statistical analyses were performed using the R program (R Foundation for Statistical Computing). Statistical significance was considered as *p* < 0.05.

## 3 RESULTS

### 3.1 Defining acquired erythrocytosis

**FIGURE 1:**
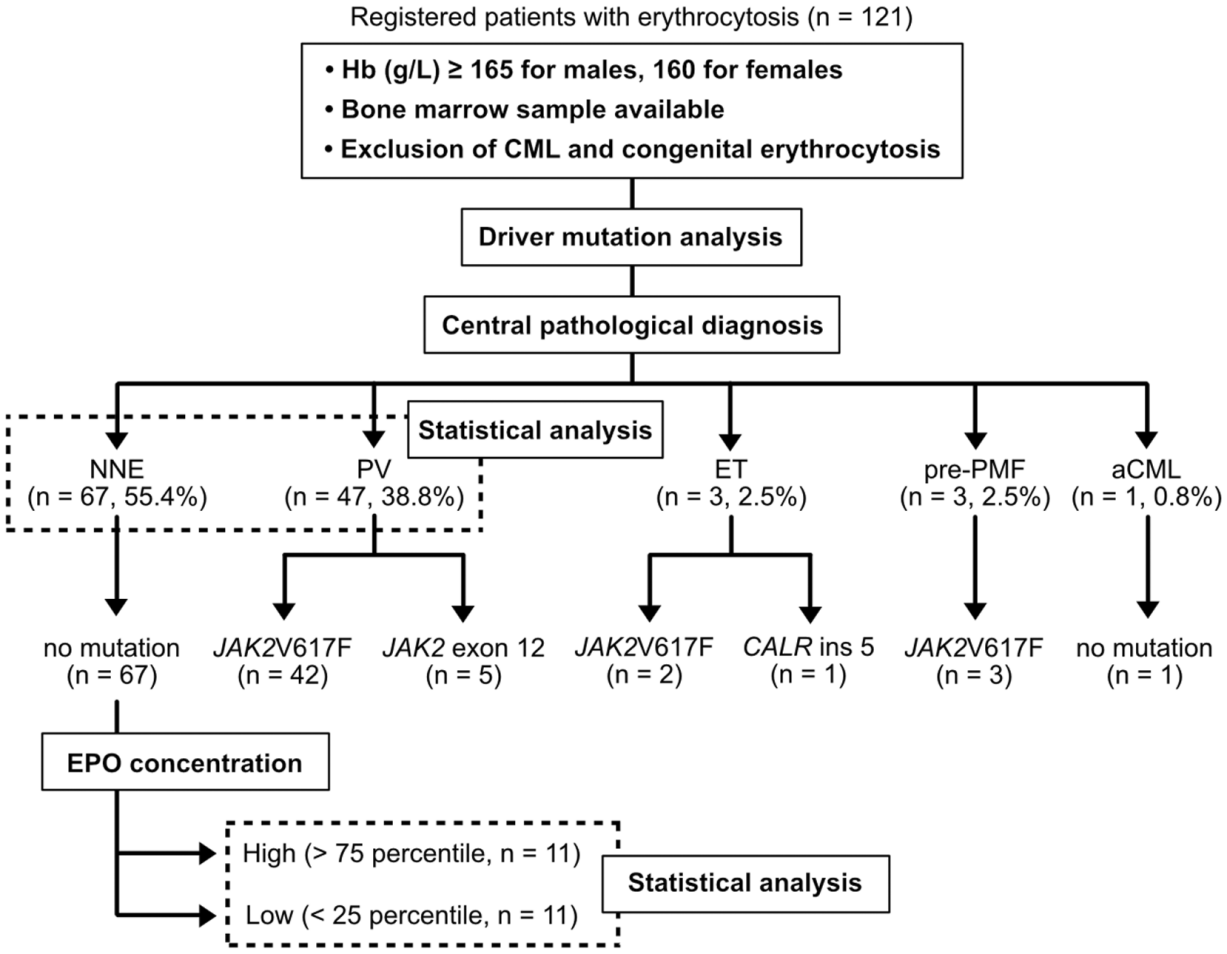
Diagram for defining acquired erythrocytosis patients. aCML, atypical CML; Hb, hemoglobin; BM, bone marrow; CML, chronic myeloid leukemia; ET, essential thrombocythemia; NNE, non‐neoplastic erythrocytosis; pre‐PMF, prefibrotic primary myelofibrosis; PV, polycythemia vera.

**FIGURE 2:**
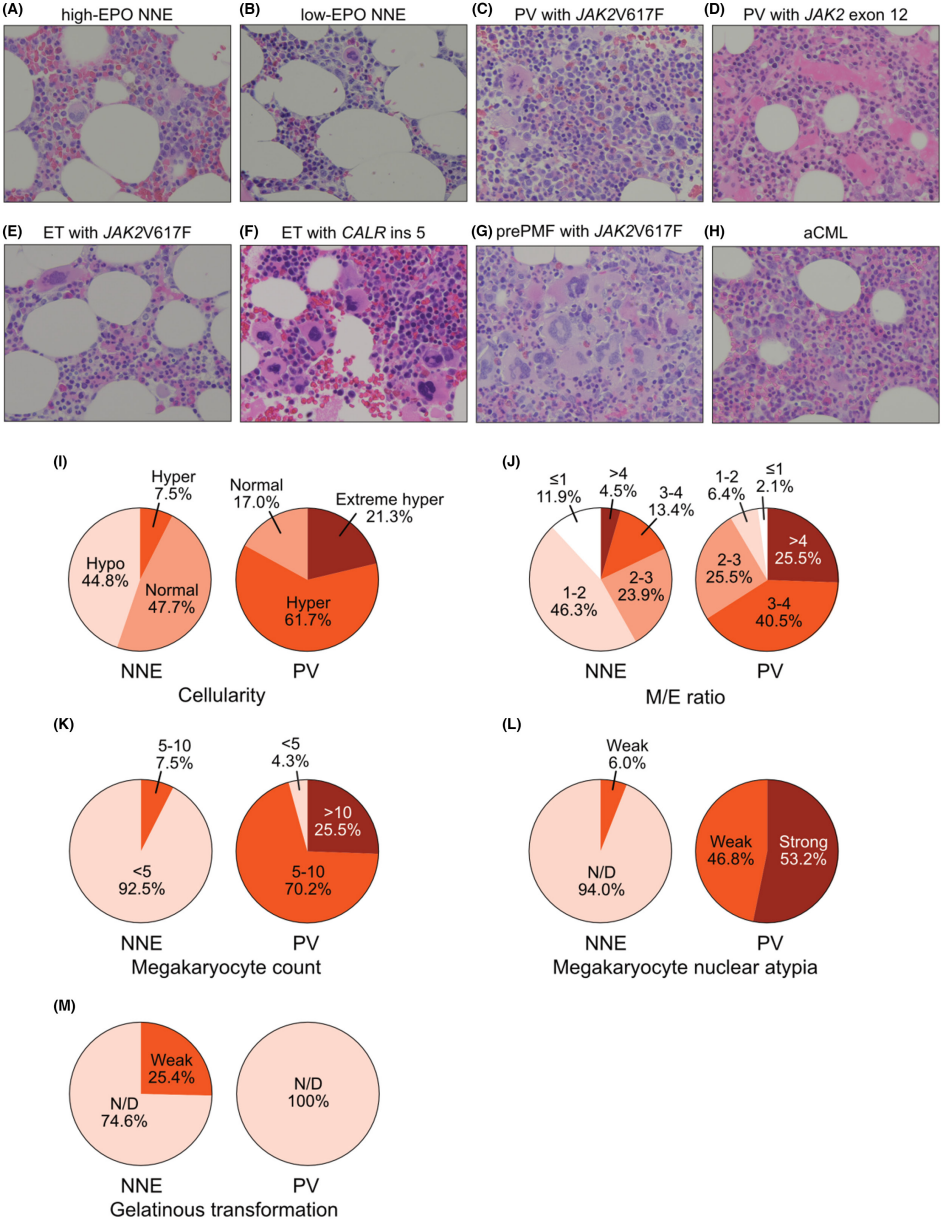
Pathological features of bone marrow biopsies of non‐neoplastic erythrocytosis (NNE) and polycythemia vera (PV) patients. Representative pathological images of NNE with high erythropoietin (EPO) levels (13.8 IU/L) (A), NNE with low EPO levels (1.7 IU/L) (B), PV with *JAK2* V617F (C), PV with *JAK2* exon 12 (D), essential thrombocythemia (ET) with *JAK2* V617F (E), ET with *CALR* ins 5 (F), pre‐primary myelofibrosis (pre‐PMF) with *JAK2* V617F (G), and atypical chronic myeloid leukemia (aCML) (H) are shown. Comparison of NNE and PV bone marrow cellularity (I), myeloid/erythroid (M/E) ratio (J), megakaryocyte count (K), megakaryocyte nuclear atypia (L), and collagenous degeneration (M). Cellularity is defined as extreme hyperplasia, >80%; hyperplasia, 60%–80%; normal, 30%–60%; and hypoplasia, <30%.

A total of 1979 individuals with suspected MPNs were screened. Among them, 121 patients fulfilled the following conditions; (1) hemoglobin (Hb) levels above the diagnostic criteria for PV (males, ≥165; females, ≥160 g/L), (2) Philadelphia chromosome‐negative, and (3) available bone marrow specimens (Figure 1). The bone marrow specimens were centrally reviewed for the evaluation of the following: cellularity, myeloid/erythroid (M/E) ratio, megakaryocyte count, megakaryocyte nuclear atypia, progenitor cell rate, gelatinous transformation, and fibrosis grade. After ruling out CML and congenital erythrocytosis, a thorough analysis of bone marrow features (Figure 2A–H) and driver mutation status (described below) were carried out, resulting in diagnosis of 67 (55.4%) patients with NNE, 47 (38.8%) patients with PV, 3 (2.5%) patients with essential thrombocythemia (ET), 3 (2.5%) patients with prefibrotic primary myelofibrosis (pre‐PMF), and 1 (0.8%) patient with atypical CML (aCML) (Figure 1).

Compared with PV bone marrow, NNE bone marrow exhibited hypo‐ to normo‐cellularity, lower M/E ratio, fewer megakaryocytes, and less nuclear abnormalities (Figure 2A–D,I–L). In the NNE group, a quarter of patients exhibited gelatinous transformation characterized by atrophy of adipose tissue, focal loss of bone marrow hematopoietic cells, and extracellular deposition of a gelatin‐like substance (Figure 2M).^13^ Despite elevated Hb levels, three ET and three pre‐PMF cases were identified based on bone marrow features such as an increase in megakaryocytes and nuclear atypia. One patient was suspected to have aCML because of a hyperplastic bone marrow with trilineage dysplasia (Figure 2H).

In the driver mutation analysis, 42 and 5 of the 47 PV patients had *JAK2* V617F and exon 12 mutation, respectively (Figure 1). Of the three ET patients, *JAK2* V617F and *CALR* ins five mutations were found in two and one patients, respectively (Figure 1). All three pre‐PMF patients harbored *the JAK2* V617F mutation. All NNE and aCML patients were negative for *JAK2* mutations (Figure 1). None of the patients had *JAK2* mutation‐negative PV.

### 3.2 Distinctive clinical characteristics of NNE

**FIGURE 3:**
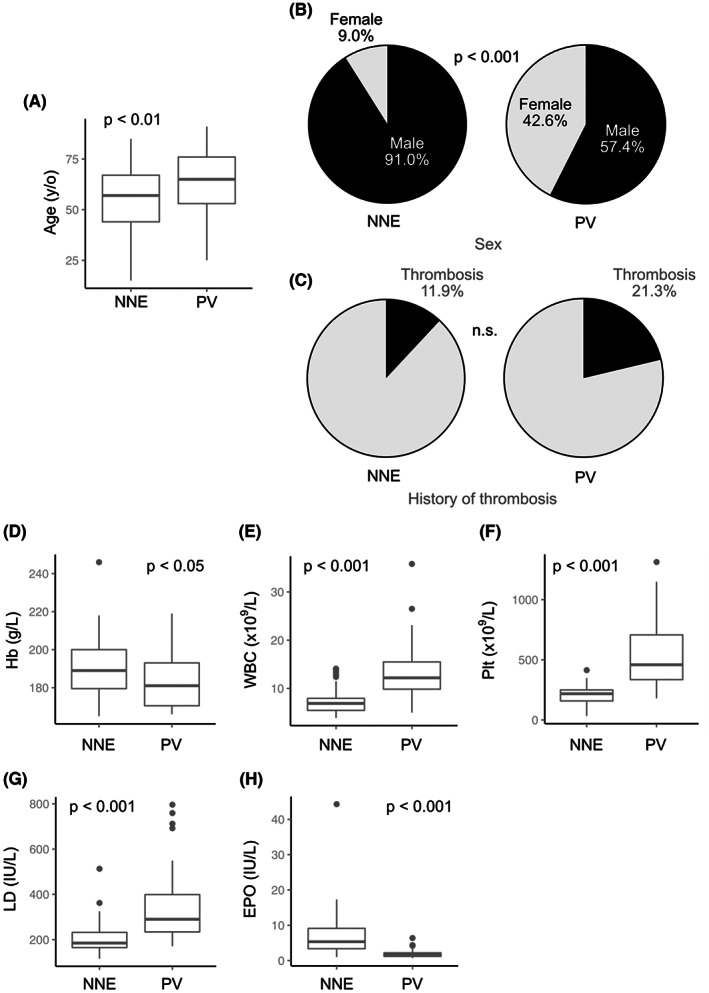
Clinical characteristics at initial diagnosis of non‐neoplastic erythrocytosis (NNE) and polycythemia vera (PV) patients. Box‐and‐whisker plots comparing age (A), pie charts comparing sex (B), and history of thrombosis (C); box‐and‐whisker plots comparing hemoglobin (Hb) levels (D), white blood cell (WBC) counts (E), platelet (PLT) counts (F), lactate dehydrogenase (LD) levels (G), and erythropoietin (EPO) levels (H) at initial diagnosis in the NNE and PV groups are shown. Thrombosis includes cerebral infarction (CI) and myocardial infarction (MI). EPO levels are evaluated only in patients with data available.

Comparison of the clinical features between 67 NNE and 47 PV patients showed the following results. NNE patients were significantly younger than PV patients (median age: 57 vs. 65 years, *p* < 0.01; Figure 3A; Table S1). In addition, the NNE group showed a male predominance (91%) which reached statistical significance when compared with the PV group (male/total: 61/67 vs. 27/47, *p* < 0.001; Figure 3B; Table S1). Splenomegaly was less frequent in the NNE group than in the PV group (10.0% vs. 32.5%). Thrombotic events, of which all were arterial in both groups, were less frequent in the NNE group than in the PV group (8 [11.9%] vs. 10 [21.3%], Figure 3C) but the difference was not statistically significant (*p* = 0.11). With respect to ischemic stroke, the prevalence was higher in the NNE group (8/67) than in the general population.^14^

Hemoglobin levels at the time of diagnosis were significantly higher in the NNE group than in the PV group, presumably reflecting the male predominance in the NNE group (Figure 3D; Table S1). White blood cell count, PLT count, and lactate dehydrogenase levels were significantly higher in the PV group compared to the NNE group (*p* < 0.001 for all of three categories, Figure 3E–G; Table S1). Platelet counts in all NNE patients were below the level required for a diagnosis of ET (450 × 10^9^/L), while the majority of PV patients showed levels higher than the diagnostic cutoff. Iron and ferritin levels were significantly lower in the PV group (*p* < 0.001; Table S1), which may be due to the neoplastic proliferation of erythroid cells. EPO was also significantly lower in the PV group (*p* < 0.001, Figure 3H; Table S1), and all but two patients in the PV group exhibited EPO levels below the lower range of normal (<4.2 IU/L). Notably, 18 (40.8%) of the 44 NNE patients with available EPO data showed low EPO levels, which is a trait that has not been previously reported in NNE.^15^, ^16^

### 3.3 Lifestyle‐related factors and pre‐existing clinical conditions in NNE

**FIGURE 4:**
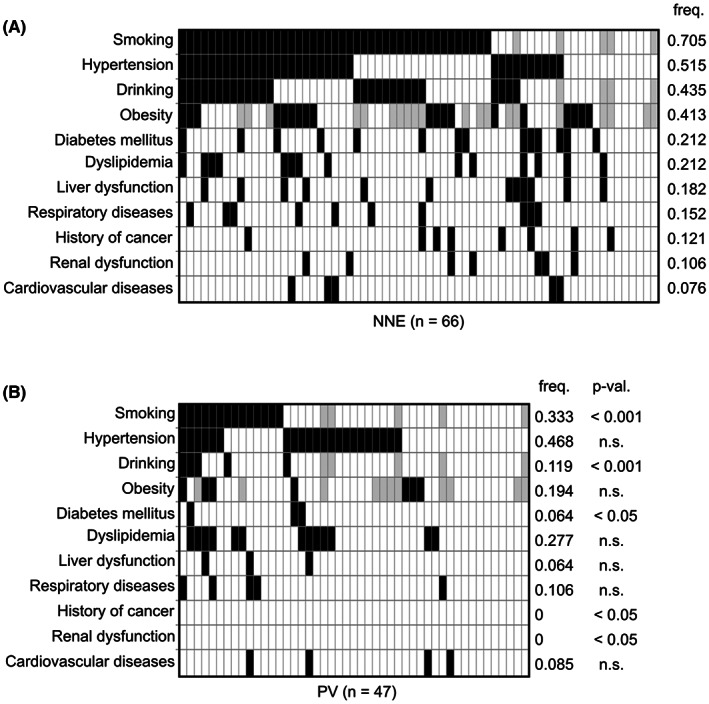
Comparison of lifestyle habits and pre‐existing clinical conditions at the time of diagnosis between non‐neoplastic erythrocytosis (NNE) and polycythemia vera (PV) patients. Heat maps illustrating lifestyle habits and pre‐existing clinical conditions at the time of initial diagnosis in NNE (A) and PV (B) patients. Black, white, and gray indicate presence, absence, and unknown status, respectively.

Comparison of lifestyle habits and pre‐existing clinical conditions are shown in Figure 4. Compared to the PV group, the NNE group showed a higher frequency of smoking (70.5% vs. 33.3%, *p* < 0.001), alcohol consumption (43.5% vs. 11.9%, *p* < 0.001), and type 2 diabetes mellitus (21.2% vs. 6.4%, *p* < 0.05). There was also a trend for higher rates of obesity (41.3% vs. 19.4%) in the NNE group. Notably, all seven patients with pronounced obesity demonstrating a BMI of ≥30 kg/m^2^ all belonged to the NNE group.

The frequencies of hypertension, dyslipidemia, and cardiovascular disease were similar between the NNE and PV groups (Figure 4). Cardiovascular diseases can cause erythrocytosis through chronic hypoxia, but no obvious increase in EPO levels was observed in patients with NNE. Diabetes mellitus, respiratory diseases, liver dysfunction, renal dysfunction, and a history of cancer were more prevalent in the NNE group (Figure 4). In both the NNE and PV groups, all patients with diabetes mellitus had type 2 diabetes. Liver dysfunction in the NNE group was mostly due to alcoholic hepatitis or fatty liver. Respiratory diseases included sleep apnea syndrome (SAS) (*n* = 3), asthma (n = 3), lung cancer (n = 2), chronic obstructive pulmonary disease (COPD) (*n* = 1), interstitial pneumonia (IP) (*n* = 1), and drug‐induced pneumonia (*n* = 1). Some of these clinical conditions may have independently induced erythrocytosis in a proportion of NNE patients. None of the above mentioned factors were found in four NNE patients (Figure 4), suggesting idiopathic erythrocytosis in these patients.

### 3.4 Clinical features associated with EPO and bone marrow features in NNE

Given that NNE includes patients with erythrocytosis caused by diverse mechanisms, we attempted to classify NNE according to biological features that might be distinct to a specific disease entity. We first studied EPO levels, which have been reported to be increased in reactive erythrocytosis.^1^ However, there was only one patient in whom the EPO was above the normal range (44.3 IU/L, normal range: 4.2–23.7 IU/L), and this patient was a heavy smoker and also obese. All eight patients highly suspected of reactive erythrocytosis due to traits such as renal artery stenosis (*n* = 1), IP and lung cancer (*n* = 1), COPD (*n* = 1), chronic heart failure (*n* = 1), SAS (*n* = 1), heavy smoking (*n* = 5), and severe obesity (*n* = 1) had normal EPO levels. These findings were consistent with some reports demonstrating increased EPO levels only in a subset of reactive erythrocytosis cases.^15^, ^17^

**FIGURE 5:**
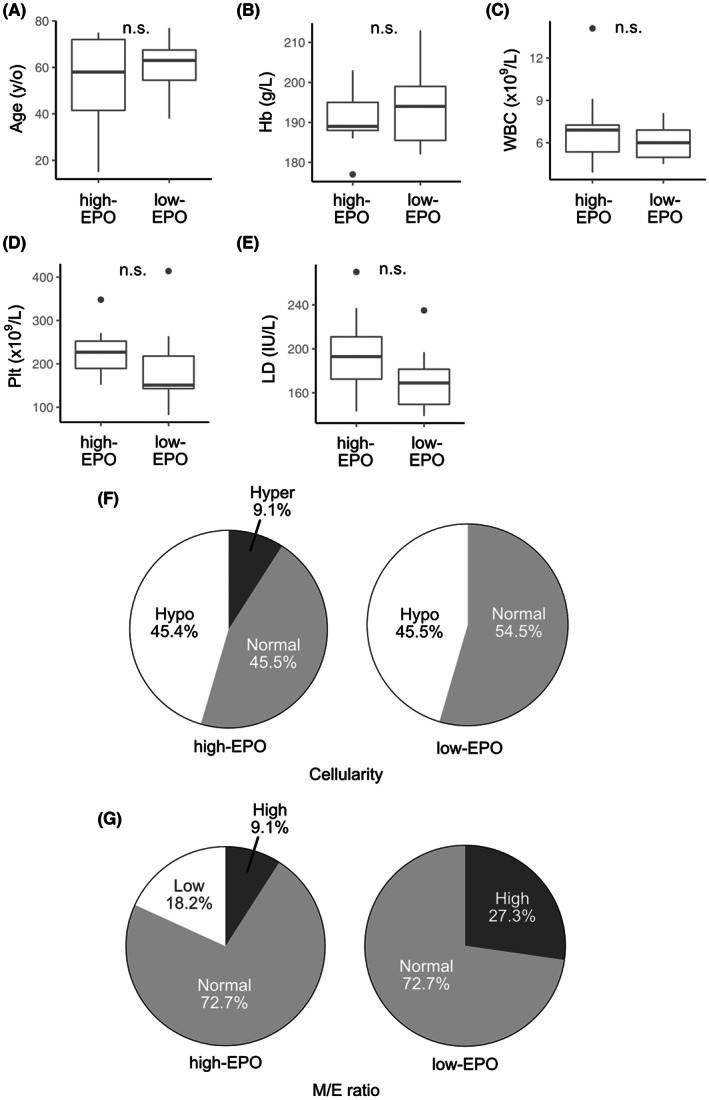
Comparison of clinical characteristics at the time of diagnosis between non‐neoplastic erythrocytosis (NNE) patients with high and low levels of erythropoietin (EPO). NNE patients whose EPO levels are 75th percentile or above (high‐EPO) and 25th percentile or below (low‐EPO) are evaluated. Box‐and‐whisker plots for age (A), hemoglobin (Hb) levels (B), white blood cell (WBC) counts (C), platelet (PLT) counts (D), and lactate dehydrogenase (LD) levels (E) are shown. Pie charts for comparison of bone marrow features concerning cellularity (F) and myeloid/erythroid (M/E) ratio (G) between high‐ and low‐EPO NNE patients are shown. Cellularity is defined as extreme hyperplasia, > 80%; hyperplasia, 60%–80%; normal, 30%–60%; and hypoplasia, <30%).

**FIGURE 6:**
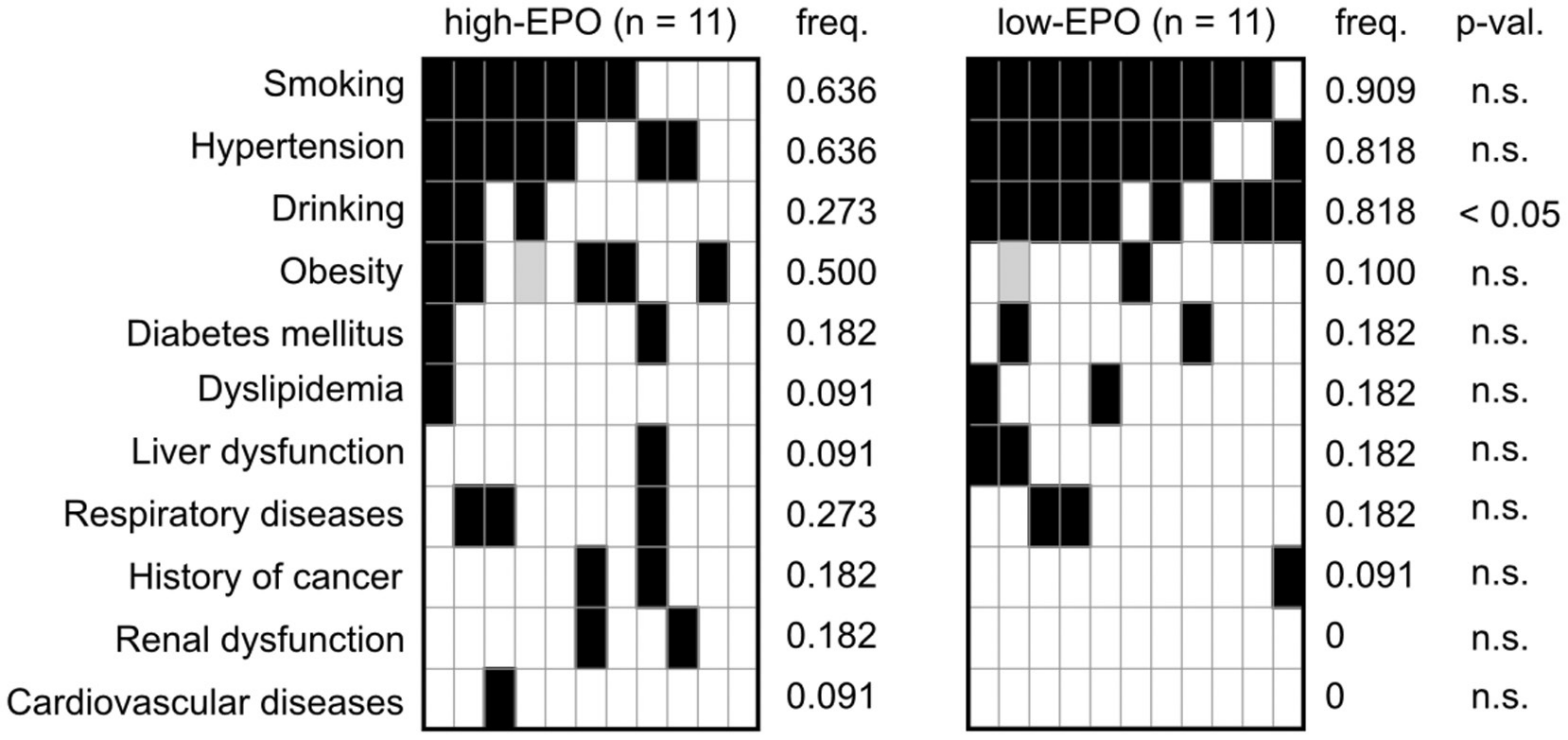
Comparison of lifestyle habits and pre‐existing clinical conditions at the time of diagnosis between non‐neoplastic erythrocytosis (NNE) patients with high and low levels of erythropoietin (EPO). NNE patients whose EPO levels are 75th percentile or above (high‐EPO) and 25th percentile or below (low‐EPO) are evaluated, and their lifestyle habits and pre‐existing clinical conditions at the time of diagnosis are illustrated as heat maps. Black, white, and gray indicate presence, absence, and unknown status, respectively.

Therefore, we compared the clinical parameters and bone marrow features between suspected NNE patients with reactive erythrocytosis who had EPO levels within the upper 25% limit (*n* = 11) and those with levels within the lower 25% limit (*n* = 11). No significant differences in clinical parameters were observed between the two groups (Figure 5; Table S2). However, the frequency of heavy drinking was significantly higher in the low EPO subgroup (81.8% vs. 27.3%, *p* < 0.05; Figure 6). Cases with cardiac disease, renal disease, and pronounced obesity with a BMI of ≥30 kg/m^2^ were not found in the low EPO subgroup.

Considering that PV presents with unique bone marrow features, we hypothesized that bone marrow morphology may be of aid in classification of distinct entities within NNE. Thus, we classified the NNE patients into three groups based on bone marrow cellularity: hypocellular (*n* = 30), normocellular (*n* = 32), and hypercellular (*n* = 5). However, comparison of clinical parameters between the first two groups that included most NNE patients showed no significant difference in any of the parameters (Figures S1 and S2). These results showed that patients with NNE could not be further classified according to bone marrow pathology.

### 3.5 *CREB3L1*mRNA in PLT is detected in PV patients but not in NNE patients

**FIGURE 7:**
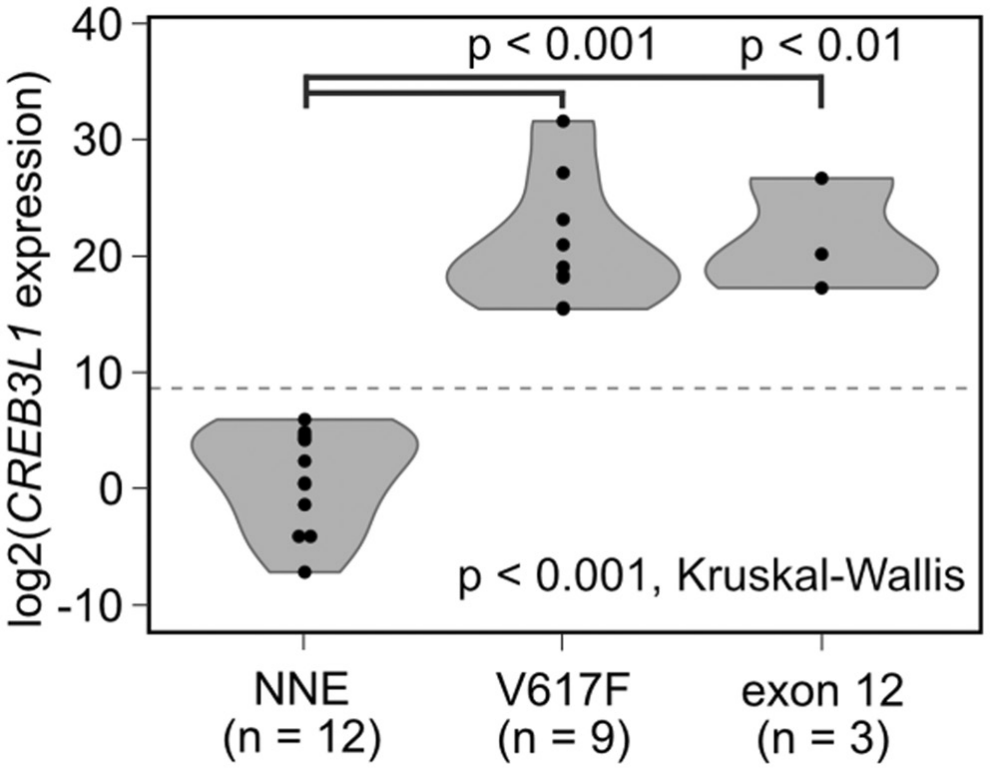
Comparison of *CREB3L1* expression levels in PLT RNA between PV and NNE patients. The levels of *CREB3L1* expression in PLT RNA are shown for NNE (*n* = 12) and PV (*n* = 12) patients. NNE, non‐neoplastic erythrocytosis; PLT, platelet; PV, polycythemia vera.

We recently reported that *CREB3L1* expression in PLT mRNA is a reliable diagnostic marker for MPN.^12^ Thus, we examined *CREB3L1*mRNA in PLT obtained from 12 NNE patients and 12 PV patients (9 patients with V617F and 3 patients with exon 12 mutations). *CREB3L1*mRNA expression was detected in all PV patients but in none of the NNE patients studied (Figure 7), confirming the diagnosis based on bone marrow pathology.

## 4 DISCUSSION

In the present study, PV, NNE, and other hematopoietic malignancies were strictly diagnosed by MPN driver mutation analysis and central review of bone marrow pathology. Compared to PV patients, NNE patients were predominantly male and had normal hematological parameters aside from Hb and hematocrit levels. Also, NNE patients presented with multiple lifestyle‐related factors and/or clinical conditions that potentially cause erythrocytosis. Although the NNE group included patients highly suspected of having reactive erythrocytosis, only one case exhibited an EPO level above the higher range of normal. This highlights a difficulty in defining reactive erythrocytosis by elevation of EPO levels. Approximately half of NNE patients exhibited EPO levels below the lower range of normal, and many of these patients showed an association with heavy drinking. Given that thrombotic events were frequent not only in PV but also in NNE patients, and also because many NNE patients presented with pre‐existing clinical conditions that are contributive to thrombosis, it can be concluded that NNE patients also need clinical attention for the prevention of thrombosis.

All PV patients were positive for *JAK2* mutations, suggesting that *JAK2*‐negative PVs are very infrequent, if they exist at all. Some patients were found to have hematological malignancies other than PV, further proving the importance of bone marrow pathology in the diagnosis of acquired erythrocytosis. A subgroup of NNE patients had low EPO levels equivalent to PV, highlighting the risk of using low levels of EPO as a diagnostic marker for *JAK2*‐negative PV without a careful bone marrow examination.

Recently, we reported that *CREB3L1*mRNA overexpression is specifically observed in PLT of MPN.^12^ In the current study, although the number of cases analyzed was small, *CREB3L1* in PLT mRNA accurately discriminated PV from NNE, demonstrating the high potential of *CREB3L1* as a reliable biomarker for the diagnosis of MPN.

Although it would be presumed that NNE should include patients with reactive erythrocytosis and increased levels of EPO, only one NNE patient in this study had EPO levels above the higher end of normal range. This may be because, in the real‐world setting, bone marrow biopsy is rarely performed in *JAK2*‐negative patients presenting with high levels of EPO, because these patients would be strongly suspected to have reactive erythrocytosis. Furthermore, although elevated EPO levels in patients with reactive erythrocytosis due to renal artery stenosis or hypoxia were expected, most of these patients in our cohort presented with normal EPO levels. This is consistent with a previous report demonstrating that elevated EPO levels are in actual uncommon in patients with reactive erythrocytosis, and they reported that only 19% of these patients had increased EPO levels.^17^ Thus, it can be concluded that EPO levels cannot be utilized for a definitive diagnosis of reactive erythrocytosis. In addition, further classification of NNE is currently not possible, because volume analysis of circulating red blood cells is not routinely available.

A subgroup of NNE patients exhibited EPO levels below the lower range of normal, which was associated with alcohol consumption. This is contrary to a previous finding that alcohol consumption was associated with an increase in EPO levels in rodents.^18^, ^19^ In addition, smoking, which was also highly frequent among the patients, has been reported to be associated with a decrease in EPO levels.^20^

Approximately 40% of the NNE patients had EPO levels below the lower limit of normal range. This could be because NNE patients who underwent bone marrow evaluation may be selected according to the low levels of EPO. The proportion of patients with EPO levels below the normal range was significantly (*p* < 0.05) higher in NNE patients with bone marrow examination (18 out of 44) than in patients who were suspected of having NNE without bone marrow examination (30 out of 131) based on Hb above the diagnostic criteria for PV and *JAK2* V617F mutation negativity. Although it is still possible that not all patients with EPO levels above the normal range were included in our cohort, the percentage of those with low level of EPO was much higher than expected. Lupak, et al. reported that of 63 patients with secondary erythrocytosis (corresponding to NNE in the current study), 4 patients (6.3%) had low EPO levels.^21^ On the other hand, Shaw et al. reported that 15 (20.8%) out of 72 patients with secondary erythrocytosis had low EPO levels.^22^ Although these two groups did not describe in detail the method of EPO measurement, the difference in the ratio of low EPO levels may be explained by the different measurement methods. Chemiluminescent enzyme immunoassay (CEIA) is in general considered to be more accurate than radioimmunoassay, and thus CEIA was adopted for measuring serum EPO levels in this study.

The incidence of thrombotic events at the time of diagnosis tended to be higher in the PV group when compared to the NNE group, but the difference was not significant (*p* = 0.18). Thrombotic events were found to be more frequent also in the NNE group when compared to normal subjects (11.8% vs. 3.1%^14^). This is consistent with a previous report that compared the frequency of thrombotic events at the time of diagnosis and found no difference between patients with secondary erythrocytosis and PV.^17^ We tried to define an association between thrombotic event and pre‐existing conditions in the NNE group; however, no conclusive evidence was obtained, which is likely due to the limited number of thrombosis cases in our cohort. Although further analysis with a larger cohort is required to define the risk factors for thrombosis, the incidence of thrombotic events in the NNE group was presumably associated with a history of smoking, obesity, and other pre‐existing clinical conditions, some of which are likely caused by the patient's lifestyle.

In summary, this study clarifies the clinical characteristics of NNE and PV, the two groups that account for the majority of acquired erythrocytosis. Most of the NNE patients had reactive and relative erythrocytosis, and a small proportion were suspected to have idiopathic erythrocytosis. No *JAK2*‐negative PV cases were found, but a subset of NNE patients presenting with low EPO levels were discovered. Thus, low EPO levels may not be reliable as generally considered for supporting the diagnosis of PV. The majority of NNE patients harbored lifestyle‐related factors and/or pre‐existing clinical conditions that were risk factors for thrombosis. Furthermore, the frequency of thrombotic events in NNE patients was similar to that of PV patients. These findings imply that prevention of thrombotic events in NNE patients is similarly important as that in PV patients, and improvement of lifestyle and pre‐existing conditions should be more vigorously sought in NNE patients.

## AUTHOR CONTRIBUTIONS

**Yosuke Mori, Marito Araki, Soji Morishita**, and **Norio Komatsu:** Conception and design. **Yosuke Mori, Marito Araki, Soji Morishita, Misa Imai**, and **Masafumi Ito:** Acquisition of data. **Yosuke Mori, Marito Araki, Soji Morishita**, and **Misa Imai:** Analysis of data. **Yoko Edahiro, Masafumi Ito, Tomonori Ochiai, Shuichi Shirane, Hajime Yasuda, Yoshinori Hashimoto, Jun Ando**, and **Miki Ando:** Interpretation of data. **Yosuke Mori, Marito Araki, Soji Morishita, Hajime Yasuda**, and **Norio Komatsu:** Writing the manuscript. **Misa Imai, Yoko Edahiro, Masafumi Ito, Tomonori Ochiai, Shuichi Shirane, Yoshinori Hashimoto, Jun Ando**, and **Miki Ando:** Revision of the manuscript. All authors approved the final version of the manuscript.

## FUNDING INFORMATION

JSPS KAKENHI grant #20H03715. Research grant from FUJIFILM Wako Pure Chemical Corporation, Fuso Pharmaceutical, Pfizer, PharmaEssentia, Perseus Proteomics, Meiji Seika Pharma, Nippon Shinyaku, Astellas and scholarship donation from Otsuka, Chugai, Kyowa Kirin, Takeda, Novartis, Sumitomo Pharma, and Bristol‐Myers Squibb.

## CONFLICT OF INTEREST

Araki and Imai are employees of Meiji Seika Pharma and Komatsu has received a salary from PharmaEssentia Japan where he is a board member. All other authors declare no conflict of interest.

## ETHICS APPROVAL STATEMENT

This study was approved by the ethics committee of the School of Medicine, Juntendo University (IRB#M12‐0895) and was conducted in accordance with the 1975 Helsinki Declaration.

## PATIENT CONSENT STATEMENT

Written informed consent was obtained prior to the use of patient samples and collection of clinical records.

## Supporting information


Data S1

## Data Availability

Data available on request from the corresponding author.
