# Supplementary material for: Clinical features of acquired erythrocytosis: Low levels of serum erythropoietin in a subset of non‐neoplastic erythrocytosis patients
Source: Cancer Med. 2022 Jul 1;12(2):1079–89. doi: 10.1002/cam4.4958 (PMC9883404; doi:10.1002/cam4.4958)
Supplement: Supplementary file 1 — Data S1 [file CAM4-12-1079-s001.docx]

**Supplemental Materials**

**Supplemental Figure 1:** **Clinical characteristics at initial diagnosis in non-neoplastic erythrocytosis (NNE) patients whose bone marrow showed hypo- and normal cellularity.**

NNE patients with hypo- and normal cellularity in the bone marrow are evaluated. Box-and-whisker plot of age (A), hemoglobin (Hb) value (B), white blood cell (WBC) count (C), platelet (PLT) count (D), LD level (E), and erythropoietin (EPO) level (F) and pie charts of sex (G) and splenomegaly (H) at initial diagnosis in NNE patients. EPO levels and splenomegaly are only evaluated for patients whose data is available.

**Supplemental Figure 2: Comparison of lifestyle habits and pre-existing clinical conditions at diagnosis between non-neoplastic erythrocytosis (NNE) patients whose bone marrow showed hypo- and normal cellularity.**

NNE patients with hypo- and normal cellularity in the bone marrow are evaluated, and their lifestyle habits and pre-existing clinical conditions at initial diagnosis are illustrated as heat maps. Black, white, and gray indicate presence, absence, and unknown status, respectively.

**Supplemental Table 1. Characteristics of the patients in the NNE and PV groups**

|  | NNE (n = 67) | PV (n = 47) | p-value |
| --- | --- | --- | --- |
| Age, median (range) | 57 (15-85) | 65 (25-91) | <0.01 |
| Male (%) | 61 (91.0%) | 27 (57.4%) | <0.001 |
| Laboratory data, median (range) |  |  |  |
| WBC (×10^9^/L) | 6.9 (3.9-14.1) | 12.2 (5.0-35.8) | <0.001 |
| RBC (×10^12^/L) | 5.89 (4.90-7.76) | 6.45 (5.12-9.70) | <0.001 |
| Hb (g/L) | 189 (165-246) | 181 (166-219) | <0.05 |
| Hct (%) | 55.1 (47.5-70.5) | 56.7 (48.5-67.2) | n.s. |
| Platelets (×10^9^/L) | 218.0 (33-414) | 459.0 (180-1313) | <0.001 |
| LD^*^ (IU/L) | 185.0 (116-513), n = 65 | 290.0 (171-796), n = 47 | <0.001 |
| Iron^*^ (×10^3^ µg/L) | 1.2 (0.38-3.11), n = 51 | 0.56 (0.15-2.51), n = 38 | <0.001 |
| Ferritin^*^ (µg/L) | 162.0 (20-1089), n = 55 | 31.5 (6-548), n = 40 | <0.001 |
| EPO^*,**^ (IU/L) | 5.4 (1.0-44.3), n = 44 | 1.8 (0.7-6.4), n = 26 | <0.001 |
| Smoking habit^*^ (%) | 43 (70.5%), n = 61 | 14 (33.3%), n = 42 | <0.001 |
| Current | 19 (31.1%) | 2 (4.8%) |  |
| Past | 12 (19.7%) | 10 (23.8%) |  |
| Unknown | 12 (19.7%) | 2 (4.8%) |  |
| Heavy drinking habit^*^ (%) | 27 (43.5%), n = 63 | 5 (11.9%), n = 42 | <0.01 |
| Splenomegaly^*^ (%) | 6 (10.0%), n = 60 | 13 (32.5%), n = 40 | <0.01 |
| History of thrombosis (%) | 8 (11.9%) | 10 (21.3%) | n.s. |
| CI | 8 (11.9%) | 8 (17.0%) |  |
| MI | 1 (1.5%) | 2 (4.3%) |  |

*Analyzed using only available data.

**A case is excluded due to regular EPO treatment.

CI: cerebral infarction, MI: myocardial infarction

**Supplemental Table 2. Characteristics of the patients with high and low EPO in the NNE group**

|  | NNE | | |
| --- | --- | --- | --- |
|  | high-EPO^*^ (n= 11) | low-EPO^*^ (n= 11) | p-value |
| Age, median (range) | 58 (15-75) | 63 (38-77) | n.s. |
| Laboratory data, median (range) |  |  |  |
| EPO median (IU/L) | 13.2 (9.5-44.3) | 1.9 (1.0-3.3) | <0.001 |
| WBC (×10^9^/L) | 6.9 (3.9-14.1) | 6.0 (4.5-8.0) | n.s. |
| RBC (×10^12^/L) | 6.1 (5.42-6.34) | 5.88 (5.48-6.71) | n.s. |
| Hb (g/L) | 189 (17.7-203) | 194 (182-213) | n.s. |
| Hct (%) | 55.1 (52.5-62) | 57.1 (53-62.1) | n.s. |
| Platelets (×10^9^/L) | 227 (152-348) | 151 (82-414) | n.s. |
| LD^**^ (IU/L) | 193 (143-270), n = 10 | 169 (139-235), n = 11 | n.s. |
| Iron^**^ (×10^3^ µg/L) | 0.93 (0.53-1.92), n = 8 | 1.63 (0.65-3.11), n = 9 | n.s. |
| Ferritin^**^ (µg/L) | 105.8 (20-671), n = 10 | 166 (34-963), n = 9 | n.s. |
| Pathology (%) |  |  |  |
| Hypercellular | 1 (9.1) | 0 | n.s. |
| Normocellular | 5 (45.5) | 6 (54.5) | n.s. |
| Hypocellular | 5 (45.5) | 5 (45.5) | n.s. |
| M/E ratio ≥3.5 | 1 (9.1) | 3 (27.3) | n.s. |
| Mgk count ≥5 | 1 (9.1) | 0 | n.s. |
| Mgk nuclear atypia | 0 | 1 (9.1) | n.s. |
| Gelatinous transformation | 5 (45.5) | 1 (9.1) | n.s. |
| History of thrombosis (%) | 0 | 2 (18.2) | n.s. |

*High EPO: NNE patients whose EPO concentration was at the ≥75^th^ percentile; low EPO: NNE patients whose EPO concentration was at the ≤25^th^ percentile.

**Analyzed using only available data.
